# Phosphorylation of Rab GTPases in the regulation of membrane trafficking

**DOI:** 10.1111/tra.12765

**Published:** 2020-10-19

**Authors:** Dieter Waschbüsch, Amir R. Khan

**Affiliations:** ^1^ School of Biochemistry and Immunology Trinity College Dublin Ireland; ^2^ Division of Newborn Medicine Boston Children's Hospital Boston Massachusetts USA

**Keywords:** effector, GDP/GTP dissociation inhibitor, GDP/GTP exchange factor, GTPase activating protein, membrane trafficking, phosphorylation, Rab GTPase, Ser/Thr kinase, tyrosine kinase

## Abstract

Rab GTPases are master regulators of membrane trafficking in eukaryotic cells. Phosphorylation of Rab GTPases was characterized in the 1990s and there have been intermittent reports of its relevance to Rab functions. Phosphorylation as a regulatory mechanism has gained prominence through the identification of Rabs as physiological substrates of leucine‐rich repeat kinase 2 (LRRK2). LRRK2 is a Ser/Thr kinase that is associated with inherited and sporadic forms of Parkinson disease. In recent years, numerous kinases and their associated signaling pathways have been identified that lead to phosphorylation of Rabs. These emerging studies suggest that serine/threonine and tyrosine phosphorylation of Rabs may be a widespread and under‐appreciated mechanism for controlling their membrane trafficking functions. Here we survey current knowledge of Rab phosphorylation and discuss models for how this post‐translational mechanism exerts control of membrane trafficking.

## Graphical abstract

Phosphorylation of Rab GTPases by serine/threonine and tyrosine kinases is emerging as a critical regulatory mechanism in membrane trafficking. This post‐translational modification can tune the strength of binding between Rabs and their interacting proteins. Phosphorylation can also influence the lifetime of active Rabs on subcellular compartments. We survey the current literature and discuss the various cellular processes connected with Rab phosphorylation.

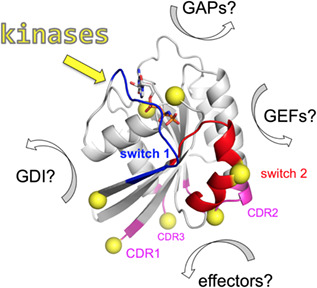

## 1 INTRODUCTION

Rab GTPases comprise the largest member of the Ras superfamily with over 60 proteins in mammalian cells.^1^ Following prenylation of C‐terminally situated cysteine residues, active (GTP‐bound) Rabs migrate to distinct subcellular compartments. The nucleotide‐bound state of Rabs is regulated by GTPase activating factors (GAPs) and GTP/GDP exchange factors (GEFs).^2^ The nucleotide sensitive switch regions (switches 1 and 2) of Rabs undergo local conformational changes that enable recruitment of GTP‐specific effectors to regulate aspects of membrane tethering/fusion, vesicle trafficking and organelle dynamics.^3^ Following hydrolysis of GTP to GDP, which is accelerated by GAPs, Rabs in their GDP form are extracted from membranes into the cytosol by GDP/GTP dissociation inhibitor (GDI).

**FIGURE 1:**
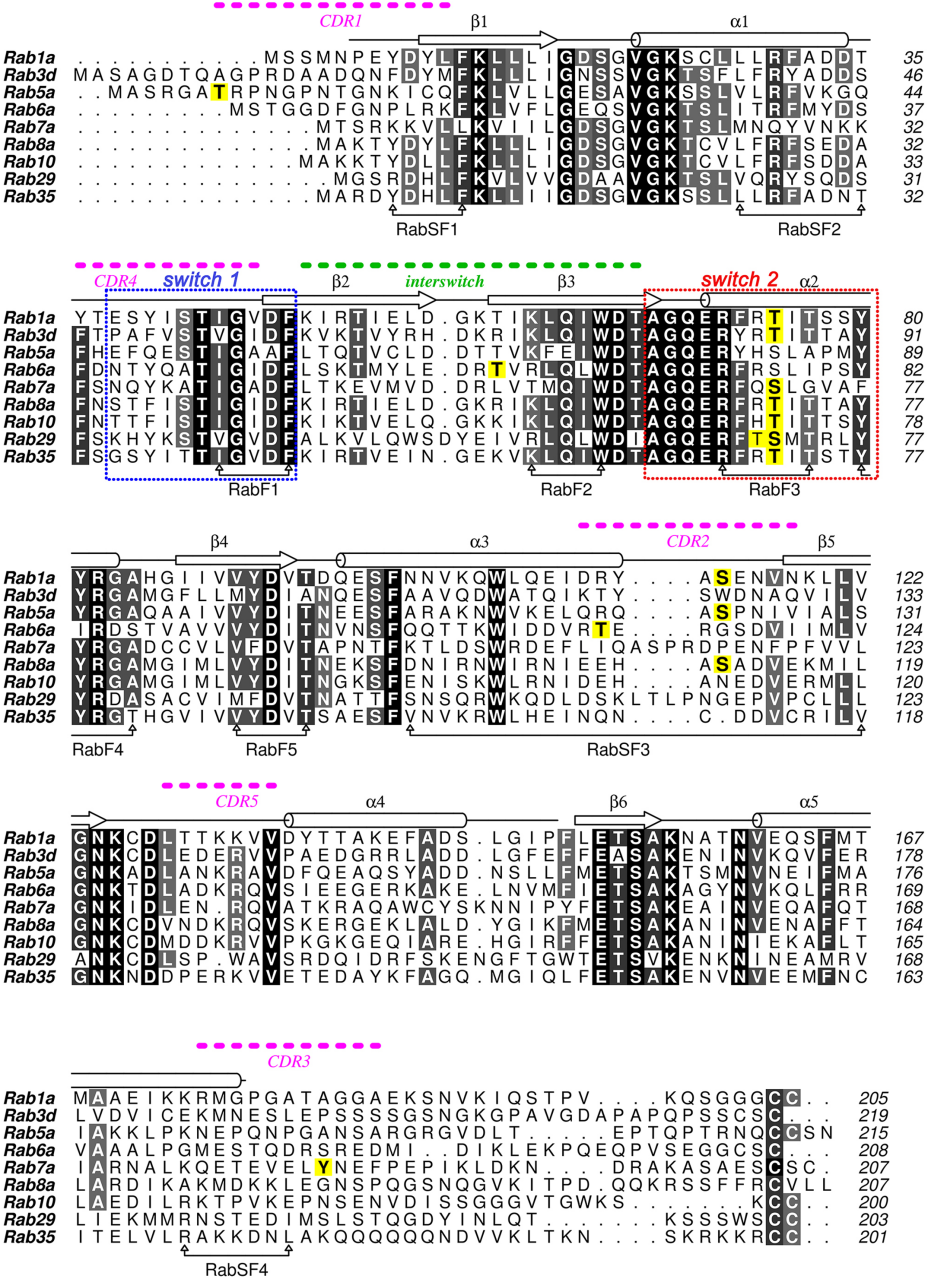
Sequence alignment of selected Rab GTPases. Phosphorylated residues have a yellow background. CDR, complementarity determining region; RabF, Rab family motif; RabSF, Rab subfamily motif. Switch 1 (blue dotted box) and switch 2 (red dotted box) are also indicated

Phosphorylation of Rab GTPases as a possible regulatory mechanism was identified in the early 1990s. Over the last 30 years, there have been intermittent reports of phosphorylation without detailed mechanistic implications. However, in the last few years, phosphorylation of Rabs has gained prominence as an additional layer of regulation that controls membrane trafficking. Phosphorylation of Rabs can alter the GTP/GDP cycle of activity by modulating the activity of GAPs, GEFs, effectors, accessory proteins, as well as the intrinsic rate of GTP hydrolysis and GTP/GDP affinities. The G protein fold comprises a 6‐stranded mixed β‐sheet flanked by 5 α‐helices is conserved in Rabs. Despite a broadly conserved fold, sequence motifs termed Rab family (RabF) and Rab subfamily (RabSF) are associated with distinct Rab functions.^4^ Rab interacting proteins (GAPs, GEFs, GDI) differentially recognize these motifs to regulate the cycle of Rab activity. These motifs overlap with complementarity determining regions (CDR1‐CDR5) that were identified as contributors to the specificity of effector recruitment.^5^ The majority of known phosphorylation sites localize to RabF/RabSF and CDR regions within the G domain (Figure 1), and therefore the modifications are probably to impact the functions of Rabs.

### 1.1 Phosphorylation of Rabs by tyrosine kinases

There are few documented cases of tyrosine phosphorylation of Rab GTPases (Table 1). When overexpressed in cultured cells, Rab24 has been shown to be phosphorylated at either Y17 and/or Y172.^6^ Phosphorylation of Y17 may affect intrinsic GTPase activity of Rab24 which is very low compared to other Rabs.^7^ Rab7 is phosphorylated at Y183 by Src kinase following activation of cells by epidermal growth factor (EGF).^8^ Phosphorylated Rab7 is unable recruit its effector RILP, thus causing enhanced Akt signaling because of the inability to degrade EGF receptor through the endocytic pathway. In a strikingly similar scenario, phosphorylation of two tyrosine residues of K‐Ras by Src kinase stalls several steps in the GTP/GDP cycle.^9^ The post‐translational modification of K‐Ras occurs adjacent to switches 1 and 2, altering the conformation of the switch regions and subsequently impairing effector interactions. Finally, Rab34 is phosphorylated by Src kinase at Y247 within the C‐terminal hypervariable region (HVD, residues 218‐259).^10^ Rab34 is localized to membrane ruffles and its overexpression is associated with aggressive breast cancer.^10^ Phosphorylation stimulates β3 integrin recycling and pulldowns using the C‐terminus of the integrin fused to glutathione S‐transferase (GST) reveal interactions with phospho‐Rab34. Although the molecular details of the interactions have not been identified, the tyrosine modification in the HVD may mediate direct interactions with β3 integrin.

**TABLE 1:** Phosphorylation of Rab GTPases and roles in trafficking

Rab	Phosphosite (Kinase)	Function	Ref.
Rab1, Rab8, Rab14	S123 (PKC, PKA, CaM kinase)	Unknown (silk moth Rabs, *Bombyx mori*)	48, 49, 50
Sec4p (yeast)	S8, and possibly S11, S201, S204 (Cdc5p)	Cell cycle, exocyst interactions	34, 35
Ypt11	S77, S158–S159	Mitochondrial inheritance	37
Rab1	T75 (TAK1), T75 (LRRK2), S114 (PINK pathway)	Cell cycle, innate immunity	11, 32, 51, 52
Rab3	unknown residues (PKC) stimulation; T86 (LRRK2)	Unknown	12, 20, 22, 51
Rab4	S204 (p34^cdc2^)	Membrane attachment during cell cycle	11, 14
Rab5	S123 (Rab 5b, p34^cdc2^; Rab5a, ERK1), T7 (PKCε)	Endosomal trafficking, T cell migration	15, 53
Rab6, Rab6c^16^	Predicted T61 or T113 (PKC)	Platelet activation, presenilin‐dependent APP processing	12, 16, 17
Rab7a, Rab7b	S17, S23, T34, T40, T64, S72, Y78, Y88 (enzymes unknown)	Subcellular localization in *Paramecium octaurelia*	54, 55
Rab7	S72 (LRRK1, TBK1, TAK1), Y183 (c‐Src)	EGFR trafficking, platelet activation, autophagy/mitophagy	13, 28
Rab8	S132 (PKC)	Bombyx and Drosphila; hormone secretion *(Periplaneta Americana*)	50, 56
Rab8	T72 (LRRK2), S111 (PINK pathway)	LRRK2 signaling, regulation of ciliogenesis, centrosomal cohesion	12, 20, 22, 23, 32, 51, 57, 58
Rab9	S179 (Ulk1)	Mitophagy impairment in ischemia	59
Rab10	T73 (LRRK2)	LRRK2 signaling, cilia formation, sonic hedgehog signaling	22, 51, 57
Rab11	S177 (PKC)	Regulation of vasoconstriction	60, 61
Rab12	S106 (LRRK2)	Phospho‐dependent binding to RILPL1/2, unknown function	20,22
Rab13	S111 (PINK pathway)	Unknown	32
Rab23	Possibly T150 (conserved as T172 in Rab37)	Response to cCMP stimulation, cellular differentiation, proliferation	62
Rab24	Y17 and Y172 (c‐Src, possibly other kinases)	Y17 (P‐loop) may impart low GTPase activity, unknown function	6, 63
Rab29	T71, S72 (LRRK2)^12^	LRRK2 signaling pathway	20, 57, 64, 65
Rab34	Y247 (c‐Src)	Cell motility in breast cancer cells, β3‐integrin recycling	10
Rab35	T72 (LRRK2)	α‐Synuclein propagation	20, 22, 66
Rab37	T172 (PKCα)	Exocytosis and metastasis, lung cancer cell motility	67
Rab43	T82 (LRRK2)	Unknown	20, 22

### 1.2 Phosphorylation of Rabs by serine/threonine kinases

There are widespread reports of Ser/Thr phosphorylation of Rabs dating as far back as 1991.^11^ In the first report of Rab phosphorylation, Rab1a and Rab4 were found to be phosphorylated by mitosis‐specific p34^cdc2^.^11^ Subsequently, thrombin‐induced platelet activation was found to result in phosphorylation of Rab3b, Rab6 and Rab8.^12^ These early studies suggested that phosphorylation led to distinct subcellular distribution of the Rabs which could impact membrane trafficking processes. In a modern follow‐up to these early studies, an in‐depth study of the phosphoproteome has integrated mass spectrometry data and literature guided cell physiological networks to profile signaling upon activation of collagen receptor GPVI.^13^ Although this paper is currently in press and the data are not available until publication, their findings suggest the involvement of >40 Rab GTPases, GEFs, GAPs and effectors. The role of TAK1‐mediated phosphorylation of Rab7 (S72) in endolysosomal maturation is particularly emphasized from this study. Here, several recent examples are highlighted as models for how phosphorylation may modulate the membrane trafficking functions of Rab GTPases (Table 1).

#### 1.2.1 Phosphorylation of Rab1, Rab3, Rab4, Rab5 and Rab6

A conserved Ser/Thr motif in switch 2 of Rab1 and Rab3b is a target of LRRK2 (see below), although no function has yet been ascribed to this modification. Similarly, reversible phosphorylation of Rab4 at S204 in the hypervariable C‐terminal domain (HVD) has been demonstrated.^14^ The protein products of the three isoforms of Rab5 (Rab5a, Rab5b and Rab5c) are differentially phosphorylated at a conserved serine (S123) by extracellular‐regulated kinase 1 (ERK1). Rab5a is particularly susceptible to ERK1, and it is also a target of PKCε in migrating T cells.^15^ Platelet activation leads to phosphorylation of Rab6c by protein kinase C. This modification affects its membrane association in fibroblasts^16^ and increases the affinity of Rab6c for GTP in vitro.^17^ In these preceding studies, the functional significance of phosphorylation remains to be determined in more detail. However, a common theme appears to be a distinct subcellular distribution upon phosphorylation, with possible links to the cell cycle and/or cytoskeletal dynamics.

#### 1.2.2 LRRK2 phosphorylation of Rabs

LRRK2 is a Ser/Thr kinase that is associated with inherited and sporadic forms of Parkinson disease.^18^ The enzyme is functionally associated with numerous aspects of post‐Golgi trafficking including in neuronal cells including autophagy, ciliogenesis and mitochondrial dynamics.^19^ Insight into LRRK2 functions has progressed significantly with the finding that a subset of small GTPases are physiological substrates of the enzyme.^20^, ^21^ LRRK2 phosphorylates Rab3a/3b/3c/3d, Rab8a/8b, Rab10, Rab12, Rab29, Rab35 and Rab43 at a conserved Ser/Thr residue in their switch 2 α‐helix.^20^ The best characterized downstream pathway involves pRab8a and pRab10. Phospho‐specific effectors that include RILPL1 and RILPL2 are recruited by pRab8a/10 and regulate the length and number of cilia in model cell lines.^22^, ^23^ RILPL1/2 are adaptor proteins, and RILPL2 also binds to the globular tail domain (GTD) of myosin Va.^24^ Hyperphosphorylation of Rab8a/10 by mutants of LRRK2, which are inherited in an autosomal dominant fashion, lead to a block in ciliogenesis and aberrant centrosome formation that may be associated with Parkinson disease.^23^, ^25^, ^26^ LRRK1 kinase bears approximately 26% sequence identities to LRRK2 and a similar domain organization. Recently, LRRK1 phosphorylation of Rab7 at Ser72 (switch 2) has been shown to promote interactions with RILP.^27^ Interestingly, Ser72 also appears to be a target for the kinase TBK1 (TANK binding kinase 1).^28^ TBK1 phosphorylation is dependent upon Parkin, an E3 ubiquitin ligase that is associated with inherited Parkinson disease.^29^, ^30^ TBK1 phosphorylation of Rab7 is associated with a decrease in recruitment of ATG9‐positive vesicles to damaged mitochondria and a defect in mitophagy.^28^ The connections between Parkinson disease, Rab7 and kinases TBK1/LRRK1 require further investigation.

#### 1.2.3 Phosphorylation of Rab8 by the PINK1 pathway

Autosomal recessive mutations in the mitochondrial kinase PINK1 are associated with Parkinson disease.^31^ The PINK1 signaling pathway directly leads to the phosphorylation of a S111 of Rab8a.^32^ However, the kinase responsible for this post‐translational modification has not yet been identified. The site lies in the α3/β5 loop of Rabs that is variable in sequence and conformation, and is situated relatively distant from the switch region. Rab1b, Rab8 and Rab13 are modified by phosphorylation at an equivalent serine residue. Despite no significant conformational changes induced by phosphorylation of S111, this post‐translational modification leads to the inability of LRRK2 to phosphorylate T72 of Rab8a.^33^ These studies suggest cross‐talk between the LRRK2 and PINK1 pathways associated with Parkinson disease.

#### 1.2.4 Phosphorylation of yeast Rabs

Sec4p is the yeast homolog of human Rab8/13 and regulates exocytosis. Using a genomic kinase screen, the activity of polo‐like kinase Cdc5p was correlated with phosphorylation of S8 in Sec4p.^34^ Phosphorylation of S8 is cell cycle dependent and negatively regulates Sec4p interactions with the exocyst component.^34^, ^35^ Ypt11 is the yeast homolog of human Rab11 and regulates mother‐to‐bud transport of various organelles during cell division. Ypt11 facilitates membrane trafficking through its interactions with the GTD of Myo2p.^36^ Although little is known about the role of specific Ypt11 phospho‐sites and associated kinases, recent work suggests that Ypt11 is at least partly phosphorylated.^37^ In this paper, a model of Ypt11 functions suggests that phosphorylation may regulate its activity through degradation and subsequent availability in cells. Although not a Rab protein, a strikingly similar scenario has been observed for Vac17p, a vacuolar adaptor for myosin mediated organelle transport.^38^ During cell division, phosphorylation of Vac17p is necessary for release from Myo2p and subsequent degradation through a ubiquitin pathway. In summary, emerging studies of yeast trafficking suggest that Rab phosphorylation may play an important role in cell division.

### 1.3 Phosphorylation hotspots within the G protein fold

**FIGURE 2:**
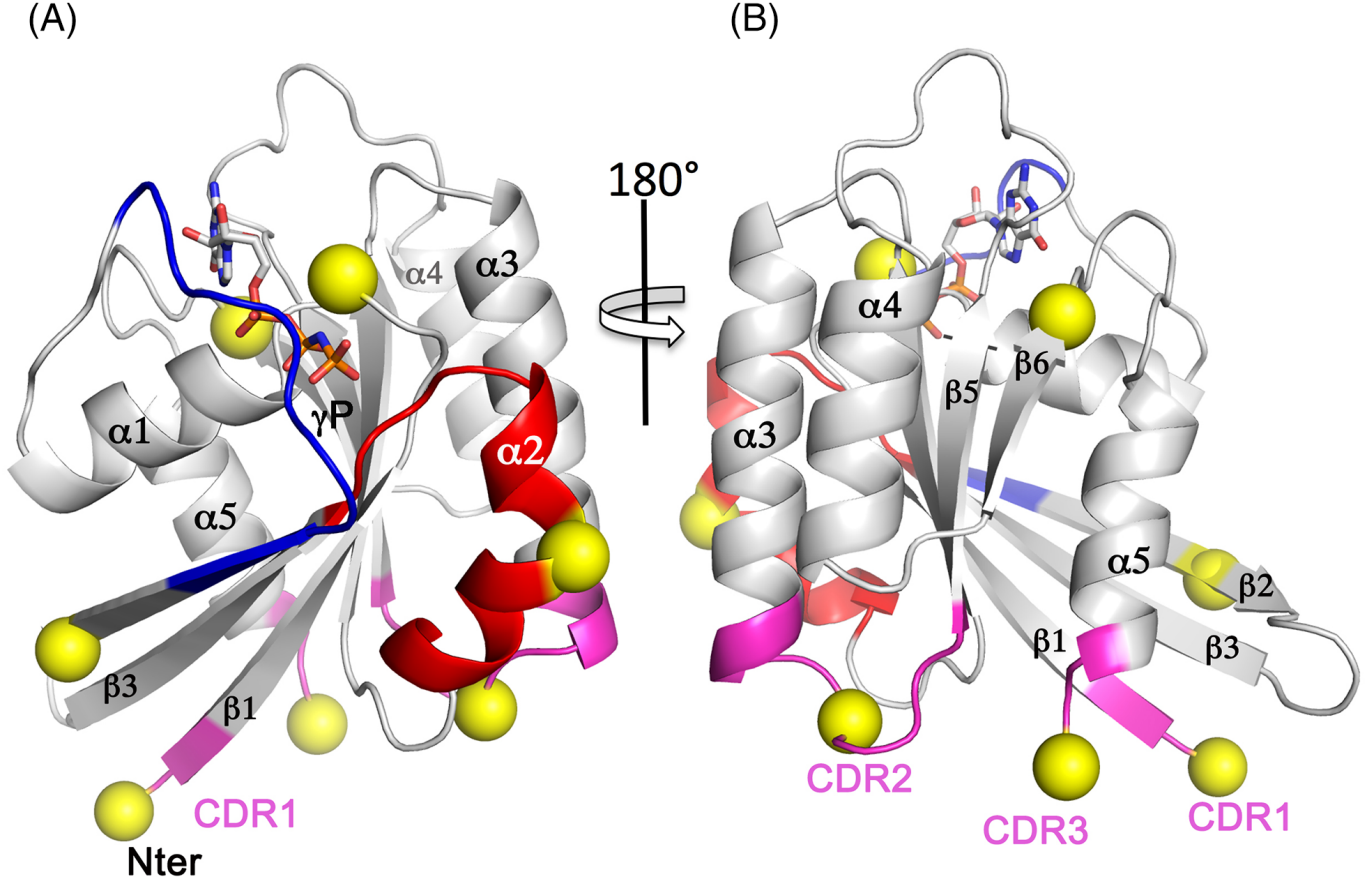
Mapping of phosphorylation sites onto the structure of Rab GTPases. The active GTP form of Rab5 (PDB code 1tu3,^47^) provides a generic template for the Rab family fold. The sites of mutations from the sequence alignment are mapped onto the template as yellow spheres. Color coding is the same as the sequence alignment. A, A view towards the switches 1 and 2 regions. B, A view 180° from the previous orientation. The three CDRs cluster together, and along with the switch 2 α‐helix, they are a hotspot for phosphorylation

**FIGURE 3:**
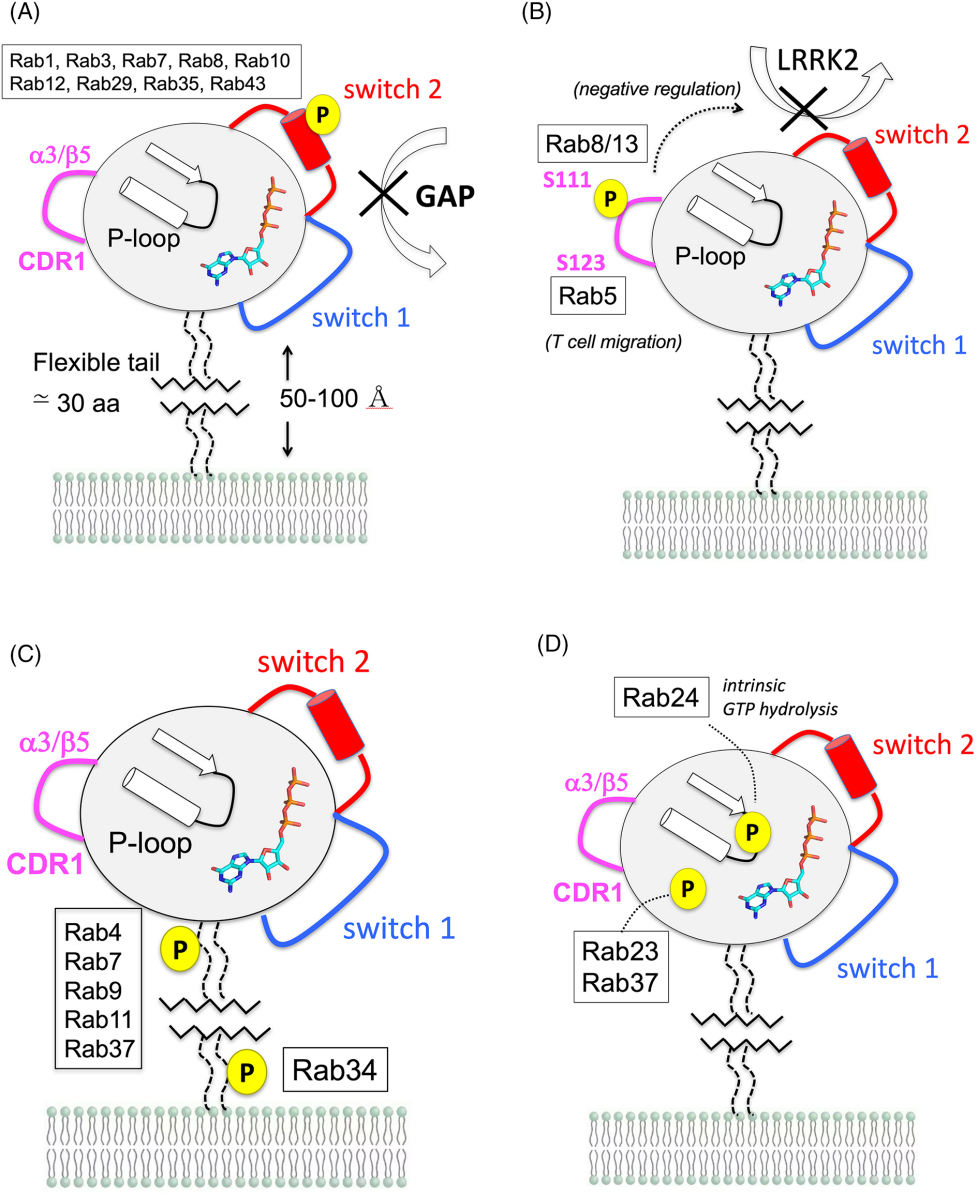
Models of the effects of phosphorylation on the functions of Rab GTPases. A, Phosphorylation of a Ser/Thr residue in switch 2. The modification inhibits GAP activity while selectively increasing/decreasing the strengths of interactions with effector proteins. B, Modification of the α3/β5 loop antagonizes the catalytic activity of LRRK2. C, A hotspot for phosphorylation is observed in the HVD, immediately following the last helix (α5). A less common membrane proximal site is also observed for Rab34. D, Phosphorylation sites near the nucleotide binding pocket, which may affect intrinsic GTP hydrolysis or affinities. The HVD is considered flexible and may extend between 50 and 100 Å from the lipid bilayer^39^

The sites of phosphorylation from Table 1 are mapped onto the conserved G protein fold to provide a structural context (Figure 2). Rab5a is used as a generic template and phospho‐sites (Ser/Thr, Tyr) are displayed as yellow spheres. The switch 2 α‐helix is a hotspot for kinases, along with the CDR1, CDR2 and CDR3. The various phosphosites are clustered into 4 common epitopes based on their positions within the G protein fold (Figure 3). We use this simplified rendering to propose that common epitopes may have similar functional consequences for Rabs. For example, phosphorylation of switch 2 is probably to prevent recognition by GAPs and prolong active Rabs on the membrane (Figure 3A). In contrast, the α3‐β5 loop is targeted by only a few kinases (Figure 3B) with distinct effects (Rab8/13 vs Rab5).

The cluster of phospho‐sites immediately following the α5 helix are intriguing (Figure 3C). Rab6 interacts with GCC185 through the HVD,^39^ but this is an exception among the Rab family. Generally, modification of the HVD by phosphate is unlikely to affect interactions between Rabs and effectors. However, phosphorylation of this region may have effects on the interaction with GDI. The HVD includes a “C‐terminal interacting motif”^40^, ^41^ that is recognized by GDI and its paralog, Rab Escort Protein (REP). Furthermore, the length and sequences of the HVDs of yeast Rabs (Ypt1, Ypt32) influence the recognition and efficiency of their GEFs, TRAPPII and TRAPPIII.^42^ Therefore, it is possible that phosphorylation in the HVD could regulate the extraction and reinsertion cycle of Rabs by modulating the interactions with GDIs and GEFs. In contrast to the “membrane distal” epitope immediately after α5, a membrane proximal site is observed in Rab34 (Y247). Phosphorylation of Rab34 may regulate its interactions with integrin β3, possibly by reducing direct Rab34: integrin interactions to promote recycling of the receptor back to the membrane.^10^ Finally, two distinct epitopes are observed for Rab24 and Rab23/37 (Figure 3D). Rab24 is phosphorylated at a tyrosine in the P‐loop, while Rab23/37 are phosphorylated at a threonine site in the loop connecting β6‐α5, relatively close to the guanine ring of GTP (Rab23/37). Apart from influencing the intrinsic GTP hydrolysis of Rab24,^6^ the effects of phosphorylation on these epitopes is unknown. Tyrosine phosphorylation also appears to be dispensable for the role of Rab24 in autophagy.^43^

## 2 CONCLUDING REMARKS

Phosphorylation of Rab GTPases at Ser/Thr and tyrosine residues adds a dimension of regulation to their roles in membrane trafficking. In the context of infection and immunity, irreversible post‐translational modification of Rabs by intracellular pathogens has been characterized as a mechanism to subvert the host trafficking machinery. For example, the Legionnaires' disease protein DrrA targets Rab1a for AMPylation at Tyr77 in switch 2, resulting in a constitutively active form that is insensitive to cellular GAPs.^44^ The same bacteria possesses AnkX, an enzyme that performs phosphocholination of Rab1 and Rab35.^45^ In contrast, cellular phosphorylation of Rab GTPases is more subtle in terms of chemistry, and may be reversible through the action of protein phosphatases. Recently, PPM1H phosphatase has been shown to counter the LRRK2 signaling cascade through dephosphorylation of pRab8a and pRab10.^46^ In summary, many of the phospho‐Rabs in Table 1 have unknown mechanistic implications, poorly defined roles in trafficking, and the specific kinase remains a mystery. One challenge for cell biologists is that recombinant expression of phospho‐Rab mimics, such as glutamate or aspartate mutants, may not be useful tools. For example, glutamate mimics of Rab8 are poor substrates for Rab geranylgeranyl transferase, leading to its mis‐localization.^23^ Despite this challenge, we anticipate further insight into the link between kinases, their substrate Rabs, and membrane trafficking in the coming years.

## CONFLICT OF INTEREST

The authors declare that they have no conflicts of interest.

## PEER REVIEW

The peer review history for this article is available at https://publons.com/publon/10.1111/tra.12765.
